# Prognostic Value of Triglyceride to High-Density Lipoprotein Cholesterol Ratio (TG/HDL-C) in IgA Nephropathy Patients

**DOI:** 10.3389/fendo.2022.877794

**Published:** 2022-06-20

**Authors:** Gaiqin Pei, Aiya Qin, Lingqiu Dong, Siqing Wang, Xiang Liu, Dandan Yang, Jiaxing Tan, Xiaoyuan Zhou, Yi Tang, Wei Qin

**Affiliations:** ^1^West China School of Medicine, Sichuan University, Chengdu, Sichuan, China; ^2^Department of Rehabilitation Medicine Center, West China Hospital, Sichuan University, Sichuan, China; ^3^Department of Nephrology, West China Hospital of Sichuan University, Chengdu, Sichuan, China; ^4^West China School of Public Health, West China Forth Hospital of Sichuan University, Chengdu, China

**Keywords:** triglyceride, high-density lipoprotein cholesterol, IgA nephropathy, prognosis, TG/HDL-C ratio

## Abstract

**Background:**

The triglycerides to high-density lipoprotein cholesterol (TG/HDL-C) ratio is an easy-to-use atherogenic and prognostic marker which has attracted increasing attention these days. However, whether TG/HDL-C correlate with outcomes in IgA nephropathy (IgAN) patients remains unknown. To clarify these issues, we conducted this study.

**Methods:**

A total of 1146 patients from West China Hospital of Sichuan University were retrospectively analysed between 2008 and 2018.The demographic, clinical and pathological data of all patients at the time of biopsy were collected. Then, patients were divided into the high TG/HDL group (TG/HDL ≥ 1.495, N=382) and the low TG/HDL group (TG/HDL-C < 1.495, N=764) based on the optimal cut-off value of the TG/HDL-C using receive operating curve. Cox proportional hazard models and Kaplan–Meier curves were used to evaluate the renal outcomes of IgAN.

**Results:**

The median age of the patients was 33 (26-42) years, and 44.5% were men. By correlation analysis, we found that the TG/HDL-C ratio was negatively correlated with the eGFR (r = 0.250, *P* < 0.001) but positively correlated with proteinuria (r = 0.230, *P*< 0.001), BMI (r=0.380, P<0.001) and serum uric (r =0.308, *P*< 0.001). Patients with a higher TG/HDL-C ratio tended to have hypertension [odds ratio (OR), 1.987; 95% CI, 1.527-2.587; *P*<0.001] and more severe pathologic lesions with tubular atrophy/interstitial fibrosis (OR, 1.610; 95% CI, 1.203-2.154; *P*=0.001). During a median follow-up period of 54.1 (35.6-73.2) months, a high TG/HDL ratio was strongly associated with worse renal survival in IgAN patients (log-rank: *P <*0.001). Multivariate Cox analysis demonstrated that a high TG/HDL-C ratio (HR 1.775, 95% CI 1.056-2.798; *P*=0.029) was an independent predictive marker to ESRD.

**Conclusion:**

In this study, we addressed the importance of TG/HDL-C ratio as a predictive marker for IgAN progression.

## Background

Immunoglobulin A (IgA) nephropathy (IgAN), characterized by diffusely deposited IgA in the kidneys, is the most prevalent primary glomerulonephritis and a leading cause of end-stage renal disease (ESRD), in which 20–40% of IgAN patients reach ESRD 10–20 years after the initial diagnosis (1). Recognizing risk factors of ESRD would be beneficial for to slowing the progression of IgAN.

Abnormal lipoprotein metabolism, which could lead to impaired renal function and accelerated atherosclerosis (2), is often characterized by the presence of high TG and low HDL-C in chronic kidney disease (CKD) (3). It is well known TG usually increases in the early stages of CKD and is associated with delayed catabolism. However, TG levels fluctuate substantially based on feeding status, thus limiting its utility as a predictive biomarker (4). The single HDL-C, despite the functions of anti-inflammatory and antithrombotic, remains controversial in predicting cardiovascular disease (CVD) or mortality (5). The combination of TG and HDL-C, which is the TG/HDL-C ratio, could therefore overcome the problem and has been proposed as a more practical atherogenic and insulin resistance marker (6, 7). It has attracted increasing attention for the better predictive value for CVD (6, 8) and disease prognosis such as peritoneal dialysis (7), coronavirus disease 2019 (9), type 2 diabetes (10), and CKD (11). However, whether the TG/HDL-C ratio could be another predictor of IgAN progression remains unknown. To clarify these issues, we conducted this study.

## Materials and Methods

### Patients

A total of 1449 patients from West China Hospital of Sichuan University between 2008 and 2018 were initially enrolled. patients with systemic disease, such as systemic lupus erythematosus, diabetes, Henoch-Schönlein purpura, liver cirrhosis or disorder of liver function, malignancy, etc., and without complete clinical and pathologic data were excluded in this study. Patients were followed up for at least 12 months or until study-defined endpoints were reached. Finally, 1146 adult biopsy-proven IgAN patients (age > 14 years) were enrolled. The research was in compliance with the Declaration of Helsinki and was approved by the ethical committees of West China Hospital of Sichuan University (2019-33). Informed consent was obtained from each patient or their legal guardians prior to treatment.

### Clinical Data

Patient information, including age, sex, clinical manifestations, laboratory indexes, renal pathology reports, and treatment strategies, systolic/diastolic blood pressure (SBP/DBP), body mass index (BMI) was obtained from electronic medical records. Laboratory values included 24-h proteinuria (UPRO), hematuria level (URBC), hemoglobin (Hb), serum albumin (ALB), serum creatinine (Cr), estimated glomerular filtration rate (eGFR), uric acid (UA), triglycerides (TG), total cholesterol (TC), and high-density lipoprotein cholesterol (HDL-C). The TG/HDL-C ratio was obtained by dividing the serum triglyceride level by the plasma high-density lipoprotein cholesterol level. Anemia, hypertension and hyperuricaemia was defined as described previously (12, 13). eGFR was calculated using the CKD-EPI equation (14). Renal biopsy samples were evaluated by an experienced pathologist and a nephrologist according to the Oxford classification (15)

### Treatments

All patients received optimal support treatment, including a full dose of angiotensin-converting-enzyme inhibitor (ACEI) or angiotensin receptor blockers (ARBs). Glucocorticoids and immunosuppressant therapy included cyclophosphamide (2 mg/kg daily for 3 months), mycophenolate mofetil (1-2 g daily for 6-8 months), tacrolimus (0.03-0.05 mg/kg daily for 6-8 months) or cyclosporin was used based on pathological classification and clinical severity according to the guidelines.

### Outcome Definition

The renal outcome was progression to ESRD, defined by commencement of renal replacement therapy or an eGFR <15 mL/min/1.73 m^2^.

### Statistical Analysis

Continuous variables are expressed as the means ± SDs or medians (interquartile ranges). Categorical variables were expressed as numbers and percentages (%). Student’s t test or the Mann–Whitney U test was used for continuous variables, and the χ2 test was used for categorical variables. The optimal thresholds of the TG/HDL ratio were obtained according to the highest Youden’s index using receiver operating curve (ROC) analyses. Kidney survival in each group was estimated by the Kaplan–Meier method. Univariate and multivariate Cox proportional hazard models were used to evaluate renal progression of IgAN. Three statistical models were used in analysis: model 1 (demographics + pathological features + TG/HDL-C), model 2 (demographics + clinical features +TG/HDL-C) and model 3 (demographics + clinical+ pathological features +TG/HDL-C). All the data were analysed by the software package SPSS 23.0 software package (SPSS, Chicago, IL, USA) and GraphPad Prism 8.0. Two-tailed *P*<0.05 was considered statistically significant.

## Results

### Demographic and Clinicopathological Characteristics

**Figure 1:**
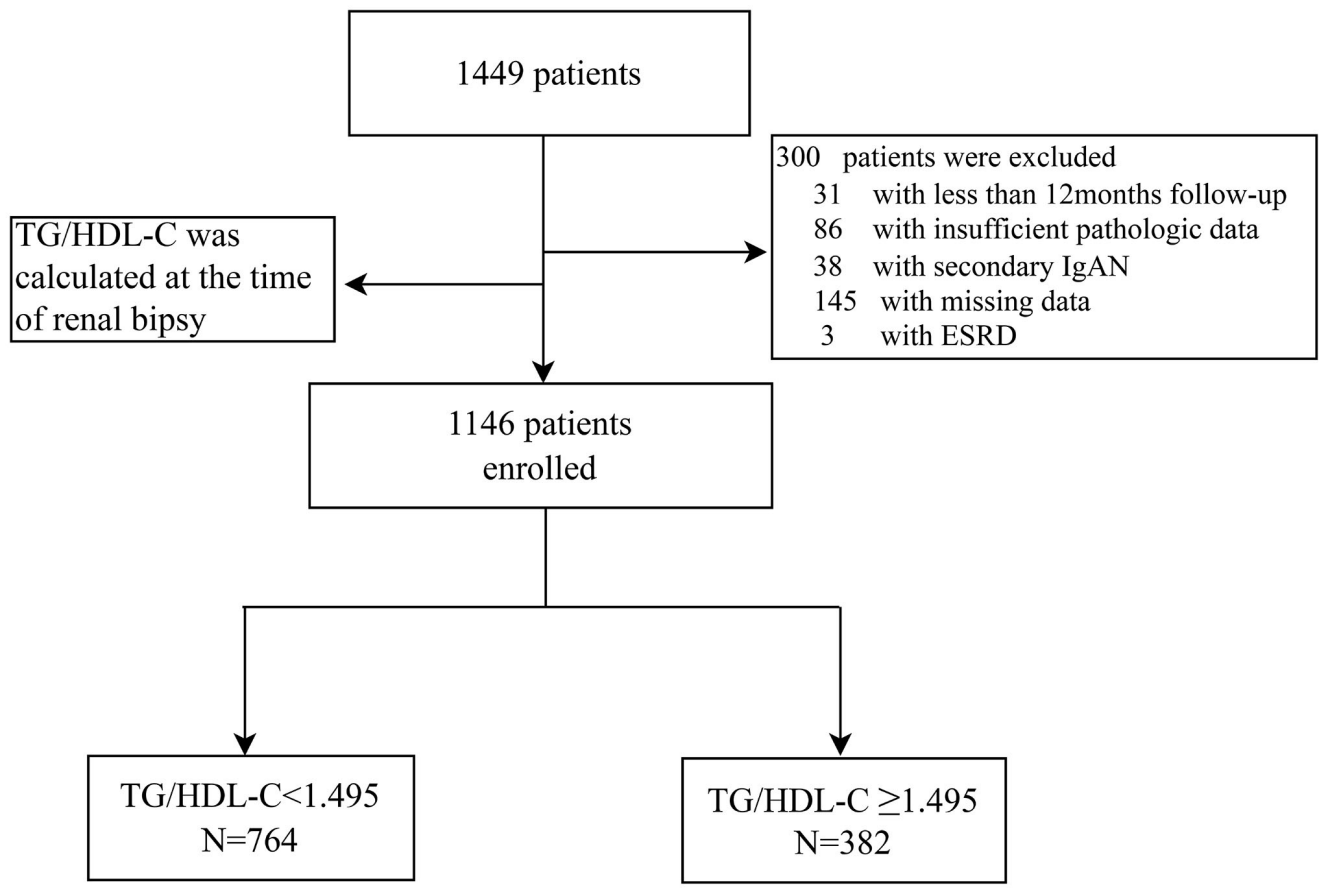
Flow diagram.

A total of 1146 biopsy-proven IgA nephropathy patients from West China Hospital of Sichuan University were finally enrolled in this retrospective study (**Figure 1**). The demographic, clinical, and pathologic characteristics of the included patients are shown in **Table 1**. The median age of the patients was 33 (26-42) years, and 44.5% were men. The median follow-up period was 54.1(35.6-73.2) months. ROC analysis revealed that the optimal cut-off TG/HDL-C ratio with which to predict the progression of ESRD in patients with IgAN was 1.495 (**Supplementary Figure 1**). Thus, according to their TG/HDL-C ratio at the time of renal biopsy, patients were divided into two groups: a high TG/HDL-C group (TG/HDL ≥ 1.495, N=382) and a low TG/HDL group (TG/HDL-C < 1.495, N=764). The median eGFR in the high TG/HDL-C and low TG/HDL-C groups was 9100.0 and 81.5 mL/min/1.73 m^2^, respectively. Interestingly, higher SBP, DBP and BMI levels were shown in the high TG/HDL-C group. Compared with the low TG/HDL-C group, patients in the high TG/HDL-C group had a higher incidence of anemia, hyperuricemia and hypertension (all *P <*0.001), a higher proportion of males (*P <*0.001), and worse renal function (*P*<0.001). Moreover, higher levels of TG (*P*<0.001), TC (*P*=0.003), UPRO (*P*<0.001), and Cr (*P*<0.001) and lower HDL-C (*P*<0.001) and URBC (*P*=0.009) levels were observed in the high TG/HDL-C group. Regarding pathological lesions, patients with a high TG/HDL-C always had mesangial hypercellularity (*P*=0.06), segmental glomerulosclerosis (*P*=0.053) and tubular atrophy/interstitial fibrosis (*P*=0.002).

**Table 1:** Demographic and clinicopathological characteristics of 1146 IgAN patients.

Parameters	Total N=1146	Group 1 (TG/HDL<1.495) N=764	Group 2 (TG/HDL ≥ 1.495) N=382	*P*
Age (year)	33 (26-42)	31 (25-41)	36 (27-44)	< 0.001
Gender (male, %)	510 (44.5)	303 (39.7)	207 (54.2)	< 0.001
HTN (%)	335 (29.2)	186 (24.5)	149 (39.2)	< 0.001
SBP (mmHg)	125 (115-138)	124 (115-137)	128 (117-139)	0.012
DBP (mmHg)	82 (75-90)	80 (74-90)	84 (76-92)	0.004
BMI (kg/m^2^)	23.1 (20.2-25.6)	22.0 (19.6-24.7)	24.9 (22.3-27.6)	< 0.001
**CKD stages (%)**				< 0.001
Stage 1	615 (53.7)	455 (59.6)	160 (41.9)	
Stage 2	290 (25.3)	178 (23.3)	112 (29.3)	
Stage 3	202 (17.6)	118 (15.4)	84 (22.0)	
Stage 4	39 (3.4)	13 (1.7)	26 (6.8)	
**Pathologic**				
M1 (%)	860 (75.1)	560 (73.3)	300 (78.5)	0.06
E1 (%)	52 (4.5)	35 (4.6)	17 (4.5)	1.00
S1 (%)	704 (61.4)	454 (59.4)	250 (65.4)	0.053
T1-2/T0 (%)	243 (21.2)	141 (18.5)	102 (26.7)	0.002
C1-2/C0 (%)	244 (21.3)	162 (21.2)	82 (21.5)	0.939
**Clinical**				
Cr (umol/L)	83.3 (65.2-109.0)	79.0 (62.0-102.0)	93.8 (72.0-126.0)	< 0.001
eGFR (mL/min/1.73 m^2^)	93.6 (66.0-117.7)	100.0 (71.8-120.1)	81.5 (54.9-105.5)	< 0.001
ALB (g/L)	40.0 (36.0-43.1)	40.1 (36.0-43.2)	40.0 (35.8-43.0)	0.694
HDL (mmol/L)	1.39 (1.11-1.73)	1.54 (1.31-1.87)	1.07 (0.89-1.26)	< 0.001
TG (mmol/L)	1.5 (1.1-2.1)	1.2 (0.9-1.5)	2.5 (2.0-3.4)	< 0.001
TC (mmol/L)	4.8 (4.1-5.7)	4.7 (4.1-5.5)	5.0 (4.2-5.8)	0.003
UPRO (g/d)	1.5 (0.8-3.0)	1.3 (0.7-2.7)	2.0 (1.0-3.5)	< 0.001
URBC (/HP)	18.0 (6.0-61.0)	19.5 (6.3-68.0)	15.0 (5.0-47.5)	0.009
Anemia (%)	161 (14.1)	88 (11.5)	73 (19.1)	< 0.001
Hyperuricemia	466 (40.7)	262 (34.3)	204 (53.4)	< 0.001
**Treatment** (%)				0.039
SC	438 (38.2)	307 (40.2)	131 (34.3)	
GC only	432 (37.7)	289 (37.8)	143 (37.4)	
IT and/or GC	276 (24.1)	168 (22.0)	109 (28.3)	
**Follow-up**				
Duration (months)	54.1 (35.6-73.2)	56.4 (36.3-75.1)	48.3 (34.7-67.6)	< 0.001
ESRD	78 (6.8)	32 (4.2)	46 (12.0)	< 0.001

Data presented as median (first-third interquartile range) or number (percentage).

SBP, Systolic Blood Pressure; DBP, diastolic blood pressure; BMI, body mass index; CKD, chronic kidney disease; M, mesangial proliferation; E, endocapillary proliferation; S, segmental sclerosis; T, tubular atrophy/interstitial fibrosis; C, crescents; Cr, creatinine; eGFR, estimated glomerular filtration rate; ALB, albumin; HDL, high-density lipoprotein cholesterol; TG, triglycerides; TC, total cholesterol; UPRO, 24 h urine protein; URBC, urinary red blood cell counts; SC, supportive care; GC, corticosteroids; IT, immunosuppressive therapy; ESRD, end stage renal disease.

### Correlation of the TG/HDL-C Ratio With Clinical Parameters and Pathological Lesions

The correlations between the TG/HDL-C levels and clinicopathological findings are illustrated in **Tables 2****,** **3**. Our results showed that TG/HDL-C was significantly negatively correlated with the eGFR (r = -0.250, *P* < 0.001) but positively correlated with proteinuria (r = 0.230, *P* < 0.001), BMI (r=0.380, *P* < 0.001) and serum uric (r =0.308, *P*< 0.001). Logistic regression analysis was conducted to analyse the relationship between TG/HDL and clinicopathologic features. The high TG/HDL-C group IgAN patients were more likely to have hypertension [odds ratio (OR), 1.987; 95% CI, 1.527-2.587; *P*<0.001] and pathologic lesions with tubular atrophy/interstitial fibrosis (OR, 1.610; 95% CI, 1.203-2.154; *P*<0.001) and segmental sclerosis (OR, 1.293; 95% CI, 1.002-1.670; *P*=0.049)

**Table 2:** Correlation between related variables and TG/HDL-C.

	Variables	Correlation coefficient (r)	*P* value
TG/HDL-C	UPRO	0.230	< 0.001**
	Hb	0.034	0.254
	UA	0.308	< 0.001**
	BMI	0.380	< 0.001**
	ALB	-0.035	0.242
	eGFR	-0.250	< 0.001**

**stands for P < 0.01.

UPRO, 24 h urine protein; Hb, hemoglobin; UA, uric acid; BMI, body mass index; ALB, albumin; eGFR, estimated glomerular filtration rate.

**Table 3:** Logistics regression models for the relationship between TG/HDL-C and kidney pathologic lesion.

	OR	95%CI	*P* value
M	1.333	0.995-1.785	0.054
E	0.970	0.536-1.755	0.920
S	1.293	1.002-1.670	0.049*
T_1-2_/T_0_	1.610	1.203-2.154	0.001**
C_1-2_/C_0_	1.016	0.753-1.370	0.919
HTN	1.987	1.527-2.587	< 0.001**

*stands for P < 0.05, **stands for P < 0.01.

M, mesangial proliferation; E, endocapillary proliferation; S, segmental sclerosis; T, tubular atrophy/interstitial fibrosis; C, crescents; HTN, hypertension.

### Renal Survival

**Figure 2:**
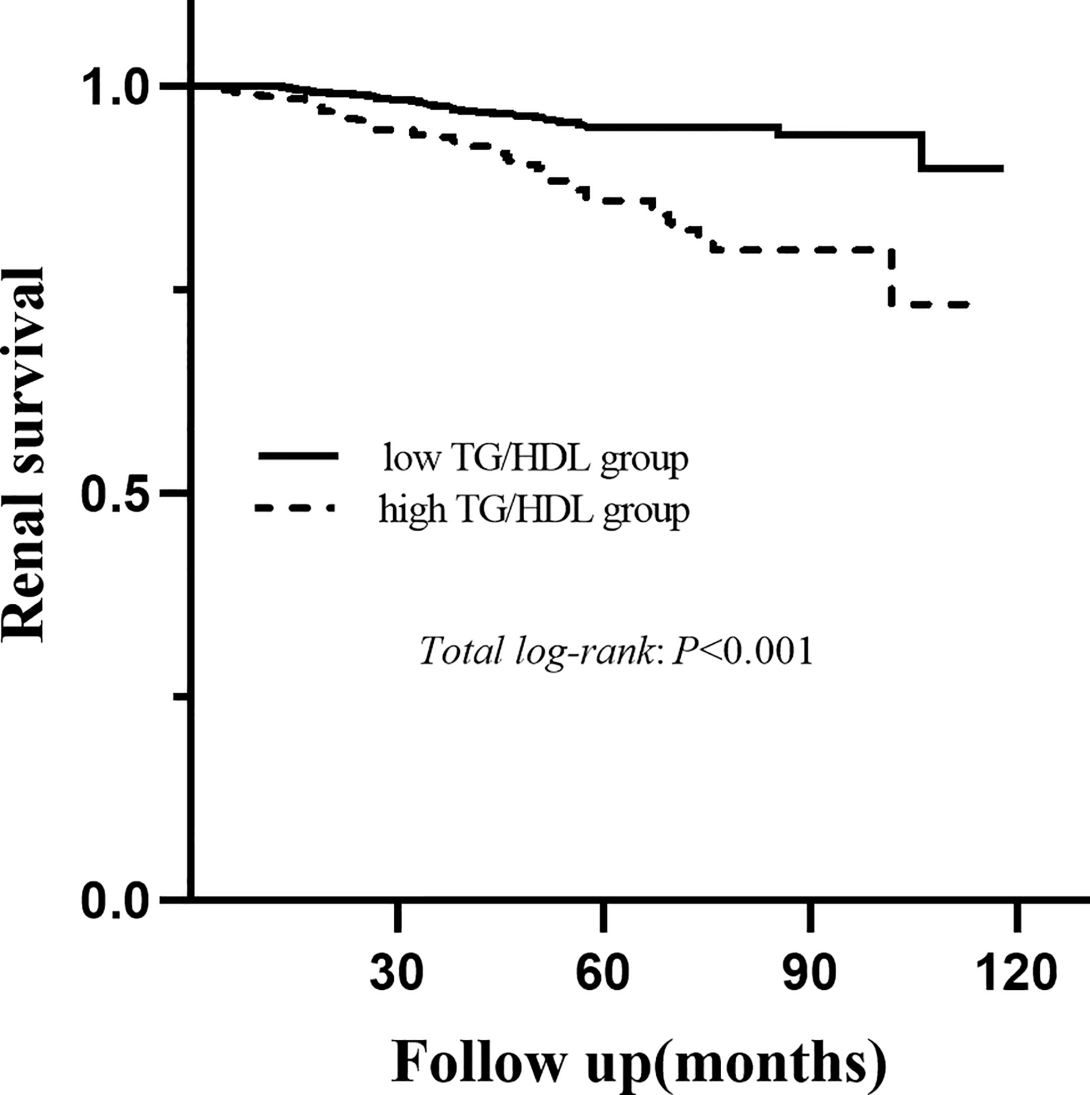
Kaplan-Meier analysis for the endpoint of ESRD stratified by the cutoff point of the TG/HDL-C.

**Figure 3:**
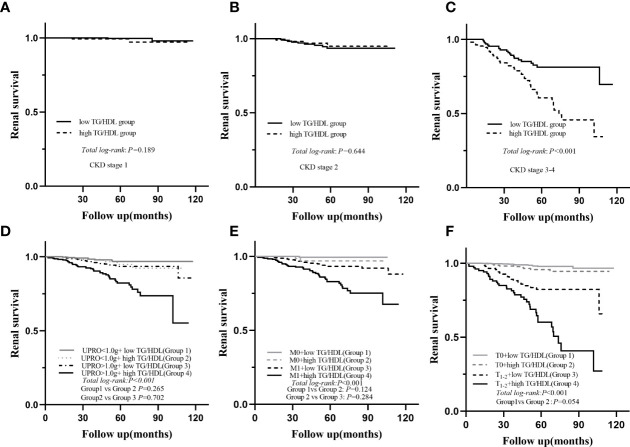
Subgroup Kaplan-Meier analysis for endpoint of ESRD; eGFR **(A–C)**, UPRO **(D)**, M **(E)**, T **(F)**; eGFR, estimated glomerular filtration rate; UPRO, 24 h urine protein; M, mesangial proliferation; T, tubular atrophy/interstitial fibrosis.

During a median follow-up period of 54.1 (35.6-73.2) months, a total of 78 (6.8%) patients developed ESRD. For renal survival, our results reveal that TG/HDL-C ≥1.495 was significantly associated with ESRD (**Figure 2**, *P* < 0.001). Subgroup analysis of mesangial hypercellularity and tubular atrophy/interstitial fibrosis, CKD stages and proteinuria for ESRD by Kaplan–Meier analysis is shown in **Figure 3**. Our results indicated that a high TG/HDL-C ratio was a risk factor for ESRD in patients with IgAN, especially patients with eGFR<60 mL/min/1.73 m^2^ (*P*<0.001) **(** **Figures 3A–C****)**, 24-hour urine protein ≥1 g/day (*P*< 0.001) **(** **Figure 3D****)**, or mesangial hypercellularity and tubular atrophy/interstitial fibrosis **(** **Figures 3E, F****)** in pathologic lesions.

### TG/HDL-C as an Independent Risk Factor for Progression of IgAN to ESRD

We performed Cox regression analyses to evaluate risk factors for ESRD in patients with IgAN, which showed that a high TG/HDL-C ratio was significantly associated with a higher risk of ESRD (HR=3.290, 95% CI: 2.093-5.173, *P*<0.001) in univariate analysis. Then, three models were used for multivariate Cox regression (**Table 4** and **Supplementary Tables 1, 2**), which indicated that high TG/HDL-C was an independent risk factor of renal endpoints (model 1: HR 4.158, 95% CI 1.970-8.775, *P*<0.001; model 2: HR 3.944, 95% CI 1.825-8.523, *P*<0.001; model 3: HR 1.775, 95% CI 1.056-2.798, *P*=0.029). Moreover, when stratified by the quartiles of baseline level of TG/HDL-C as Q1, Q2, Q3 and Q4 group (**Supplementary Figure 2**), significant difference were also shown between Q4 (highest quartiles) and other groups (Q1, Q2, and Q3)

**Table 4:** Analysis of factors associated with renal outcomes in model 3 (demographics+ clinical indicators+ pathological features+ TG/HDL-C).

Parameter	Univariate			Multivariate		
	HR	95%CI	*P* value	HR	95%CI	*P* value
high TG/HDL-C	3.290	2.093-5.173	< 0.001	1.775	1.056-2.798	0.029
male	1.902	1.213-2.982	0.005	–	–	–
Age (per year)	0.991	0.971-1.012	0.410	0.951	0.928-0.976	< 0.001
BMI (kg/m^2^)	1.048	0.956-1.149	0.319	–	–	–
SBP (mmHg)	1.033	1.022-1.044	< 0.001	–	–	–
DBP (mmHg)	1.049	1.034-1.063	< 0.001	–	–	–
M1	8.806	2.776-27.937	< 0.001	4.320	1.348-13.847	0.014
E1	2.232	1.073-4.642	0.032	–	–	–
S1	1.614	1.001-2.602	0.049	–	–	–
T_1-2_/T_0_	11.811	7.154-19.499	< 0.001	2.475	1.398-13.847	0.002
C_1-2_/C_0_	1.290	0.786-2.115	0.314	–	–	–
UPRO>1.0g	3.310	1.823-6.007	< 0.001	–	–	–
URBC>5/HP	0.947	0.559-1.605	0.840	–	–	–
Anemia	3.717	2.339-5.908	< 0.001	–	–	–
Hyperuricemia	4.457	2.778-7.573	< 0.001	–	–	–
hypoalbuminemia	2.255	1.302-3.907	0.004	–	–	–
CKD stages						
CKD 2 vs 1	6.993	2.260-21.268	0.001	6.800	2.147-21.538	0.001
CKD 3 vs 1	30.497	10.970-85.394	< 0.001	23.898	7.714-74.031	< 0.001
CKD 4 vs 1	136.066	46.960-394.247	< 0.001	63.129	19.189-207.689	< 0.001
Treatment			0.010			0.036
GC/SC	0.738	0.414-1.317	0.304	1.191	0.705-2.011	0.514
IT/SC	1.710	1.019-2.869	0.042	0.563	0.319-0.992	0.047

SBP, Systolic Blood Pressure; DBP, diastolic blood pressure; BMI, body mass index; M, mesangial proliferation; E, endocapillary proliferation; S, segmental sclerosis; T, tubular atrophy/interstitial fibrosis; C, crescents; UPRO, 24 h urine protein; URBC, urinary red blood cell counts; SC, supportive care; GC, corticosteroids; IT, immunosuppressive therapy.

## Discussion

Dyslipidemia is common in China, with a prevalence of 41.9 %, and is commonly characterized by the presence of high TG and low HDL-C in CKD (16). Recently, the combination of TG and HDL-C, the TG/HDL-C ratio, has attracted increasing attention for its better predictive power for cardiovascular events and insulin resistance than the lonely combination (17, 18). Noticeably, several studies have shown a positively relationship between the TG/HDL-C ratio and renal function decline in CKD patients (16, 18–20). However, whether TG/HDL-C has a role in IgAN progression remains unknown.

In this study, 78 (6.8%) patients developed ESRD in our 1146 biopsy-proven IgAN patients. A higher TG/HDL-C ratio in patients with IgAN was associated with more severe clinical features and pathologic lesions. Our further analysis revealed a higher serum TG/HDL-C level was an independent risk factor for the progression to ESRD (HR 1.775, 95% CI 1.056-2.798, *P*=0.029).

Previous studies have reported that the reduction in renal function in patients is related to high TG/HDL-C levels (11, 16), but no study has focused on IgAN patients. To our knowledge, this is the first study assessing the correlation between the TG/HDL-C ratio and ESRD in IgAN patients. Moreover, the TG/HDL-C ratio has a greater influence in advanced IgAN patients (eGFR< 60 mL/min/1.73 m^2^) or these with 24-hour urine protein of ≥1 g/day in our study. This might be due to the slow progression of mild renal disease, especially within a limited follow-up period (12).

Here, higher SBP, DBP and BMI levels were shown in the high TG/HDL-C group. Further analysis showed TG/HDL was positively correlated with BMI (r=0.380, *P* < 0.001). In our multivariate Cox regression analyses, BMI was not independent risk factor for renal progression. It is well known obesity often result in excessive accumulation of energy, accompanied by abnormalities in lipid parameter levels. Some studies showed it was associated with the early development of hypertension and CKD progression (21). However, emerging epidemiologic evidence indicated that obesity was not a direct and independent risk factor for IgAN (22). It was possible that high BMI indirectly accelerated the progression of IgAN by inducing metabolic syndrome on patients, in which TG/HDL-C is strongly associated with it (23). Additionally, we would like to stress that high TG/HDL-C patients tend to have hypertension and more severe renal pathologic lesions of segmental glomerulosclerosis and tubular atrophy/interstitial fibrosis. Considering that abnormalities in lipid parameter levels could accelerated atherosclerosis is plausible to believe that pathology of IgAN patients with a high TG/HDL-C level.

Abnormalities in lipid parameter levels could lead to impaired renal function and accelerated atherosclerosis (2). TG usually increases in the early stages of CKD and is associated with delayed catabolism and decreased activity of hepatic TG lipase and peripheral lipoprotein lipase. However, based on feeding status, TG levels could fluctuate substantially, thus limiting its utility as a predictive biomarker (4). HDL-C, inversely associated with outcomes, decreases in patients with CKD (11). The combination of these two markers could therefore overcome this problem and lead to a far more consistent, stable, fasting measurement of dyslipidaemia, which has indeed attracted much more attention in disease prognosis, including cardiovascular disease (6), diabetes (10), and peritoneal dialysis (7). Here, the potential mechanisms by which the TG/HDL-C ratio serves as a prognostic biomarker in IgAN patients may be as follows. TG/HDL-C is a reliable indicator of insulin resistance, which induces oxidative stress. Oxidative stress impairs the activation of nuclear factor erythroid-2-related factor-2, which protects against kidney tissue injury (16). The filtered proteins, such as fatty acids, phospholipids, and cholesterol contained in the filtered proteins (albumin and lipoproteins), could include direct toxic effects of lipids on glomerular cells and promote matrix production (3, 24). Moreover, these materials act as damage-associated molecular patterns (DAMPs) and are recognized by Toll-like receptors (TLR2 and TLR4), which can activate inflammatory responses, causing tubulointerstitial fibrosis and injury in the reabsorption process (25, 26). Additionally, further tissue injury is contributed owing to impaired HDL-mediated reverse cholesterol transport by limiting the unloading of the excess cellular cholesterol and phospholipid burden (25). Thus, in the future, recognizing the TG/HDL-C ratio as a potentially modifiable risk factor for patients may allow the utilization of preventative strategies to optimize both treatment and survival outcomes.

Some limitations warrant consideration. First, this was a retrospective study based on a single-center database. Second, the median follow-up time of 54 months was relatively short. Further multicenter validation in different ethnic populations with longer follow-ups was needed.

## Conclusion

The TG/HDL-C ratio may be considered as a prognostic biomarker in IgAN patients.

## Data Availability Statement

The raw data supporting the conclusions of this article will be made available by the authors, without undue reservation.

## Ethics Statement

The studies involving human participants were reviewed and approved by the ethical committees of West China Hospital of Sichuan University (2019-33). The patients/participants provided their written informed consent to participate in this study.

## Author Contributions

GP, AQ, SW, LD, XL, and DY collected and analysed the data. GP and AQ wrote the main manuscript text. YT, XZ and JT reviewed and edited the manuscript. WQ supervised all the work and revised the manuscript. All authors contributed to the article and approved the submitted version.

## Funding

This study is partly supported by the National Key R&D Program of China (2020YFC2006503) &(2020YFC2006500).

## Conflict of Interest

The authors declare that the research was conducted in the absence of any commercial or financial relationships that could be construed as a potential conflict of interest.

## Publisher’s Note

All claims expressed in this article are solely those of the authors and do not necessarily represent those of their affiliated organizations, or those of the publisher, the editors and the reviewers. Any product that may be evaluated in this article, or claim that may be made by its manufacturer, is not guaranteed or endorsed by the publisher.

## Supplementary Material

Supplementary Figure 1 ROC curves (AUC) of TG/HDL-C for prediction of ESRD.

Supplementary Figure 2 Kaplan-Meier analysis for the endpoint of ESRD stratified by quartile: Q1, first quartile; Q2, median; Q3, third quartile, Q4, fourth quartile.
