# Supplementary figures and images for: Prognostic Value of Triglyceride to High-Density Lipoprotein Cholesterol Ratio (TG/HDL-C) in IgA Nephropathy Patients
Source: Front Endocrinol (Lausanne). 2022 Jun 20;13:877794. doi: 10.3389/fendo.2022.877794 (PMC9251124; doi:10.3389/fendo.2022.877794)

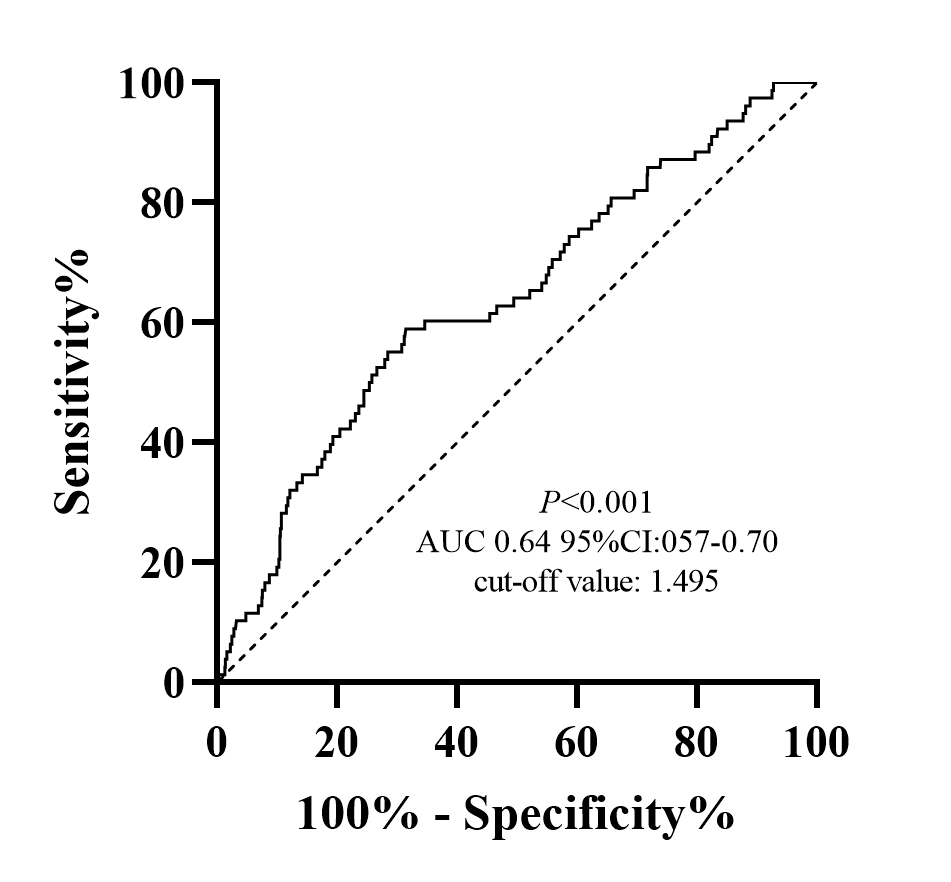

Supplement: Supplementary Figure 1 — ROC curves (AUC) of TG/HDL-C for prediction of ESRD. [file Image_1.tif]

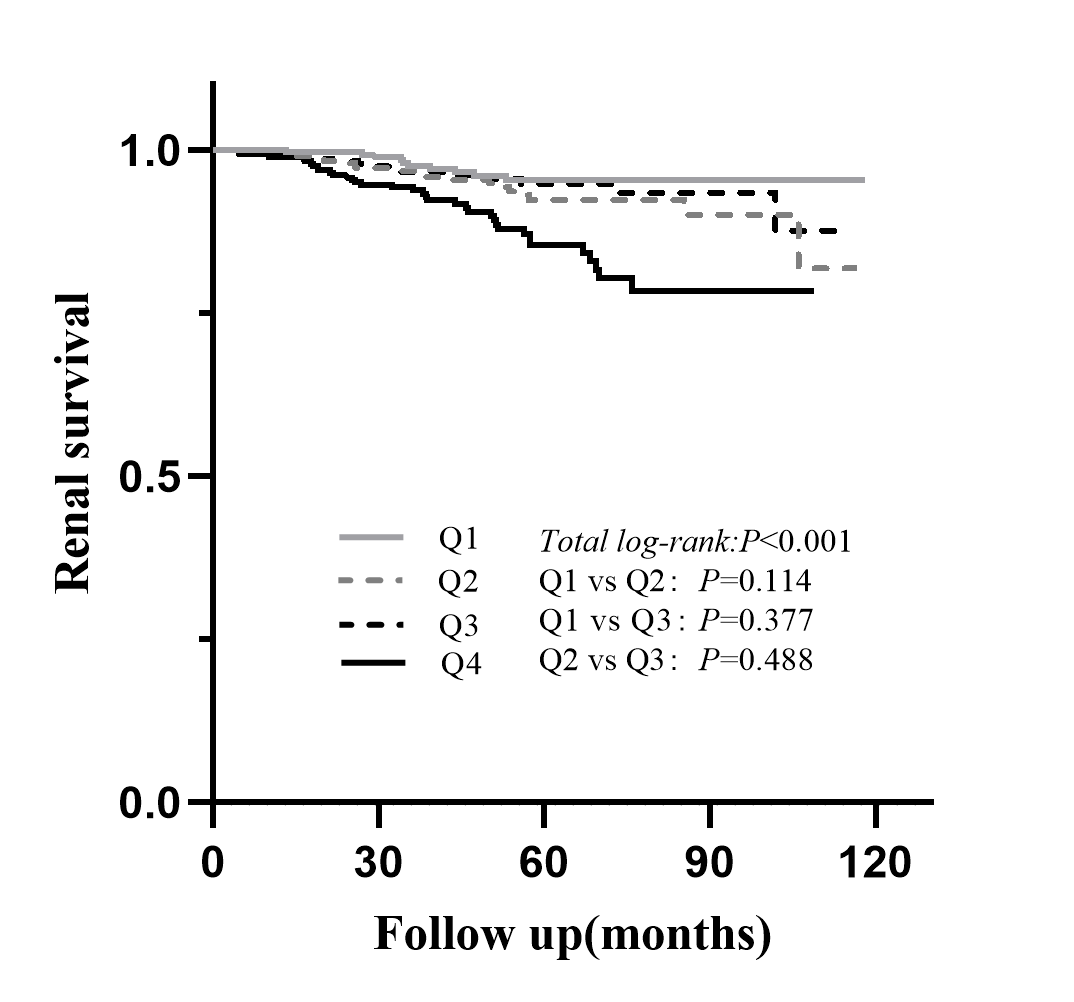

Supplement: Supplementary Figure 2 — Kaplan-Meier analysis for the endpoint of ESRD stratified by quartile: Q1, first quartile; Q2, median; Q3, third quartile, Q4, fourth quartile. [file Image_2.tif]
