# Supplementary material for: Prognostic Value of Triglyceride to High-Density Lipoprotein Cholesterol Ratio (TG/HDL-C) in IgA Nephropathy Patients
Source: Front Endocrinol (Lausanne). 2022 Jun 20;13:877794. doi: 10.3389/fendo.2022.877794 (PMC9251124; doi:10.3389/fendo.2022.877794)
Supplement: Supplementary file 3 [file DataSheet_1.docx]

**Supplementary table 1.** Analysis of factors associated with renal outcomes in model 1 (demographics+ pathological features +TG/HDL-C).

| Parameter | Univariate | | | | | Multivariate | | |
| --- | --- | --- | --- | --- | --- | --- | --- | --- |
|  | HR | | 95%CI | *P* value | HR | | 95%CI | *P* value |
| high TG/HDL-C | | 3.290 | 2.093-5.173 | <0.001 | 4.158 | | 1.970-8.775 | <0.001 |
| Male | | 1.902 | 1.213-2.982 | 0.005 | - | | - | - |
| Age (per year) | | 0.991 | 0.971-1.012 | 0.410 | - | | - | - |
| BMI (kg/m^2^) | | 1.048 | 0.956-1.149 | 0.319 |  | |  |  |
| SBP (mmHg) | | 1.033 | 1.022-1.044 | <0.001 |  | |  |  |
| DBP (mmHg) | | 1.049 | 1.034-1.063 | <0.001 |  | |  |  |
| M1 | | 8.806 | 2.776-27.937 | <0.001 | - | | - | - |
| E1 | | 2.232 | 1.073-4.642 | 0.032 | - | | - | - |
| S1 | | 1.614 | 1.001-2.602 | 0.049 | - | | - | - |
| T_1-2_/T_0_ | | 11.811 | 7.154-19.499 | <0.001 | 6.624 | | 3.114-14.090 | <0.001 |
| C_1-2_/C_0_ | | 1.290 | 0.786-2.115 | 0.314 | - | | - | - |

**Abbreviations:** SBP, Systolic Blood Pressure; DBP, diastolic blood pressure; BMI, body mass index; M, mesangial proliferation; E, endocapillary proliferation; S, segmental sclerosis; T, tubular atrophy/interstitial fibrosis; C, crescents;

**Supplementary table 2.** Analysis of factors associated with renal outcomes in model 2 (demographics+ clinical indicators+ + TG/HDL-C).

| Parameter | Univariate | | | Multivariate | | |
| --- | --- | --- | --- | --- | --- | --- |
|  | HR | 95%CI | *P* value | HR | 95%CI | *P* value |
| high TG/HDL-C | 3.265 | 2.093-5.173 | <0.001 | 3.944 | 1.825-8.523 | <0.001 |
| male | 3.290 | 1.213-2.982 | 0.005 | - | - | - |
| Age (per year) | 1.902 | 2.093-5.173 | 0.420 | 0.946 | 0.912-0.981 | 0.003 |
| BMI (kg/m^2^) | 1.048 | 0.956-1.149 | 0.319 | - | - | - |
| SBP (mmHg) | 1.033 | 1.022-1.044 | <0.001 | - | - | - |
| DBP (mmHg) | 1.049 | 1.034-1.063 | <0.001 | - | - | - |
| UPRO>1.0g | 3.310 | 1.823-6.007 | <0.001 | - | - | - |
| URBC>5/HP | 0.947 | 0.559-1.605 | 0.840 | - | - | - |
| Anemia | 3.717 | 2.339-5.908 | <0.001 | - | - | - |
| Hyperuricemia | 4.457 | 2.778-7.573 | <0.001 | - | - | - |
| hypoalbuminemia | 2.255 | 1.302-3.907 | 0.004 | - | - | - |
| CKD stages |  |  | <0.001 |  |  | <0.001 |
| CKD 2 vs 1 | 6.993 | 2.260-21.268 | 0.001 | 15.204 | 1.749-132.184 | 0.014 |
| CKD 3 vs 1 | 30.497 | 10.970-85.394 | <0.001 | 93.948 | 12.410-711.194 | <0.001 |
| CKD 4 vs 1 | 136.066 | 46.960-394.247 | <0.001 | 194.060 | 21.424-1757.790 | <0.001 |
| Treatment |  |  | 0.010 | - | -- |  |
| GC/SC | 0.738 | 0.414-1.317 | 0.304 | - | - | - |
| IT/SC | 1.710 | 1.019-2.869 | 0.042 | - | - | - |

**Abbreviations:** SBP, Systolic Blood Pressure; DBP, diastolic blood pressure; BMI, body mass index; UPRO, 24 h urine protein; URBC, urinary red blood cell counts; SC, supportive care; GC, corticosteroids; IT, immunosuppressive therapy;
